# High Intensity Functional Training (HIFT) and competitions: How motives differ by length of participation

**DOI:** 10.1371/journal.pone.0213812

**Published:** 2019-03-21

**Authors:** Allyson G. Box, Yuri Feito, Chris Brown, Katie M. Heinrich, Steven J. Petruzzello

**Affiliations:** 1 Department of Kinesiology and Community Health, University of Illinois Urbana-Champaign, Urbana, IL, United States of America; 2 Department of Exercise Science and Sport Management, Kennesaw State University, Kennesaw, GA, United States of America; 3 Department of Kinesiology, Kansas State University, Manhattan, KS, United States of America; California State University San Marcos, UNITED STATES

## Abstract

High Intensity Functional Training (HIFT) is a unique fitness method that promotes an active lifestyle and has seen exponential and continual growth over the last two decades. Motivation to exercise is likely to change over time as individuals’ motives to initiate exercise may be different than those which motivate them to maintain an exercise program. The purpose of this study was to examine the motivational factors reported by individuals who actively engage in HIFT with varying length of participation and competition levels. 737 adults (32.4 ± 8.2 years) with more than three-months of HIFT experience completed an online version of the Exercise Motivation Inventory (EMI-2) survey. Those who had greater length of participation reported more motives associated with relatedness (i.e., affiliation, competition) and enjoyment, while those with less HIFT participation were more motivated by body-related variables (i.e., weight management). Further, motivational variables (e.g., social recognition, affiliation, challenge) varied depending on whether or not individuals had competed in an online qualifier. Understanding these differences in motivation may aid in exercise promotion, initiation, and adherence, and moreover promote long-term physical and mental health benefits.

## Introduction

It is generally understood that regular physical activity positively impacts physical and mental health [1, 2]. However, most individuals have yet to adopt physical activity as a regular behavior [3, 4]. The Physical Activity Guidelines Advisory Committee [1] suggested individuals should accumulate a minimum of 150 minutes of moderate intensity aerobic exercise per week and two or more days of full-body muscle strengthening exercise to obtain improvements in various health outcomes. In 2011, Tucker and colleagues noted that approximately 60% of individuals were meeting the aerobic physical activity guidelines via self-report; however, when assessed by accelerometry, only 9.6% of those surveyed actually met these guidelines [4]. In 2015, the Center for Disease Control and Prevention suggested that over 25% of adults in the United States did not engage in any physical activity [3].

To better understand why individuals do not regularly engage in exercise (i.e., not meeting minimal recommendations), previous investigators have examined barriers and motivations to engage in various exercise forms [5]. Although many barriers have been cited, lack of motivation and time are the most common [6, 7]. Moreover, a general association has been shown between enjoyment and adherence [8, 9], suggesting that someone who enjoys participating in a specific activity would be more likely to adhere to participation in said activity. Thus, considering lack of motivation is a significant barrier to physical activity and exercise, it is important to understand an individual’s motives across exercise forms in order to encourage his or her initiation and potential maintenance of a physically active lifestyle.

Many theoretical frameworks have been developed in attempts to explain how individual’s behavior change occurs. Among these, Self-Determination Theory (SDT) [10] has become a prevalent framework for promoting physical activity and understanding exercise behavior [11, 12]. A basic proposition of Self-Determination Theory [10] is that individuals must fulfill three basic needs in order to maintain a particular behavior (e.g., exercise). These basic needs are: 1) mastery, the perceived ability to be successful/competent during a task; 2) relatedness, the perceived social network/connection between those completing the same or similar task; and 3) autonomy, the perception that task engagement is more autonomous (i.e., intrinsic) or controlled (i.e., extrinsic). Autonomous perceptions are thought to be more self-determined [10] and reflective of greater adherence to behavior. According to Deci and Ryan [10], autonomous behavior (i.e., greater self-determination) is a reflection of an individual’s behavioral regulation (e.g., management, control). The three basic forms of motivation include intrinsic motivation (a direct desire from within the individual (e.g., enjoyment), extrinsic motivation (goals that gain reward or avoid punishment), and amotivation (simply a lack of motivation). It should be noted that an individual may fluctuate on this regulation continuum at any given time, and may likely perform a particular behavior (e.g., HIFT) that is simultaneously intrinsically and extrinsically regulated/motivating [10]. Theoretically, behavior change (or adherence to a behavior) is expected to occur for individuals who achieve the basic needs of autonomy, mastery, and relatedness [6, 10].

### High Intensity Functional Training

High Intensity Functional Training (HIFT), is an exercise modality that “incorporates a variety of functional movements, performed at high-intensity [relative to an individual’s ability], and designed to improve parameters of general physical fitness (e.g., cardiovascular endurance, strength, body composition, flexibility, etc.) and performance (e.g., agility, speed, power, strength, etc.) [13], and has exponentially grown as a fitness trend over the past two-decades and continues to draw significant numbers of participants [14]. According to physical activity guidelines, individuals engaging in vigorous aerobic activities only need half the time per week (i.e., 75 minutes) to achieve positive health outcomes compared to those participating in moderate intensity activities. Considering HIFT programs could be as short as 5 minutes [15], and participants are often working above 90 percent of their maximal heart rate [16, 17], the typically shorter duration workouts may serve as a motivator to some, as lack of time has been cited as a primary barrier to physical activity [6, 7]. Even with these shorter periods of higher intensity exercise, investigators have shown positive physiological adaptations to HIFT [18–21], and workouts result in energy expenditure levels that meet ACSM’s physical activity guidelines [22, 23].

HFIT as a group exercise modality, and similar to other sports and group exercise forms, has been shown to elicit intrinsic motivation (i.e., enjoyment, challenge), competition, and relatedness factors [23–26]. However, because HIFT has been adopted by individuals across a wide range of populations (e.g., children, elderly, obese, physically disabled, elite competitors), further understanding of the motivational factors which drive individuals to initiate and adhere to this training regimen is needed.

Every year, individuals around the world engaging in HIFT have an opportunity to participate in a five-week, online competition (Online). This online competition is open to anyone who registers and provides an opportunity to compete with other HIFT participants around the world. Of those who register (over 350,000 in 2018; [27]), the top 640 participants advance to compete in a second round (Second round), and the winners obtain a final place in the final competition (Finals). The final part of the competition includes the top 80 competitors around the world (40 males, 40 females). With the exception of the workouts in the second round, each workout challenge is unique and unknown to the competitors prior to its release. With so few (<0.05%) competing in the final round, it is possible the motivation to engage in this form of exercise will vary greatly across years of experience and competitive level [27].

As HIFT continues to grow as a fitness modality, a need arises to understand the different motivational factors that influence exercise initiation and adherence to this type of exercise training, as well as the motivational differences for those who use type of training as a fitness competition platform. Therefore, the purpose of this study was to examine the motivational factors reported by individuals who actively engaged in HIFT by accounting for varying length of training and participation in different levels of competitions. It was hypothesized that: 1) those with greater length of participation in HIFT would have greater intrinsic motivation (i.e., more autonomous/self-determined) and be more driven by fitness-related factors (e.g., strength & endurance); 2) those with fewer years of HIFT participation would have greater extrinsic motivation and be more driven by body and health-related motives (e.g., ill-health avoidance); and 3) those who compete in any type of HIFT competition will have greater interpersonal motivation (e.g., social recognition, affiliation, competition) compared to health and body-related motivation (e.g., positive health, weight management, appearance) when compared to their non-competing counterparts.

## Materials and methods

### Design and participants

This study was designed to reach a large cross-sectional convenience sample of individuals participating in HIFT. Inclusion criteria required being an adult (>18 years) with more than 3-months of HIFT experience and having access to the internet. An online questionnaire was used to reach a potentially large sample of individuals. The survey, completed through an online platform (Google forms, Google, Mountain View, CA), was distributed via social media to HIFT gyms throughout the world, targeting owners and members of the community. The online application, Bitly (Bitly, Inc. New York, NY), was used to shorten the survey link and track the number of “clicks” the survey received. This allowed us to estimate the global reach and calculate a response rate for the survey without storing Internet protocol (IP) addresses from any computer, thus keeping the survey completely anonymous. All participants provided informed consent prior to beginning the survey, and the study protocol was approved by the Kennesaw State University Institutional Review Board (IRB # 13–167). All data were collected via a Google form survey and downloaded into Excel 2011 (Microsoft Co., Redmond, WA).

### Measures

The survey included several descriptive and demographic questions, along with a question regarding the length of participation the respondent had with HIFT (<6 months, 6 months-<1 year, 1–2 years, 3–5 years, >5 years). The revised 51-item Exercise Motivation Inventory (EMI-2) [28], a validated measure of motivation, was used to investigate 14 participatory motives that may drive individuals to participate in HIFT. Each subscale is comprised of 2–4 items rated on a 6-point Likert-type scale (0 = “not at all true for me”, and 5 = “very true for me”). Participants reply to statements concerning the reasons why they “personally exercise (or might exercise)”. The EMI-2 measures an individual’s reasoning for exercise by examining health-related (health pressures, ill-health avoidance, positive health), fitness-related (strength and endurance and nimbleness (e.g., agile, quick)), body-related (appearance and weight management), interpersonal (affiliation, social recognition, and competition), and psychological motives [28]. For this study, psychological motives were referred to as intrapersonal motives [29, 30] and included revitalization (e.g., invigorating, energizing), enjoyment, challenge, and stress management. These were thought to reflect an individual’s intrinsic motivations, while interpersonal factors, such as affiliation (e.g., social connectedness; also considered an intrinsic motivator, see Deci & Ryan, 2008), social recognition, and competition, reflected extrinsic motivations [28]. The EMI-2 participatory motives, except for Revitalization (Chronbach’s α = 0.656) and Health Pressures (α = 0.465), had acceptable reliability (Chronbach’s α_s_ = 0.748-.929) for this study.

### Statistical analysis

In an attempt to reduce possible Type I errors [31], multivariate analysis of variance (MANOVA) were conducted utilizing Dunn-Bonferroni method during post-hoc analyses. Overall MANOVA’s revealed an age (Wilk’s λ = 0.308, F(46,690) = 1.301, p< 0.001, η^2^_p_ = 0.081) and sex (Wilk’s λ = 0.855, F(2, 735) = 4.200, p< 0.001, η^2^_p_ = 0.075) effect on the data, and thus age and sex variables were controlled for in subsequent MANOVAs to examine differences in the distribution of the 14 subscales based on the participant’s HIFT experience (i.e., length of participation) and level of competition (i.e., Online, Second Round, Finals). Partial eta-squared (*η*^*2*^_*p*_) was used as an estimate of effect size (0.02 = small effect, 0.13 = medium effect, 0.26 = large effect; [32]. Post-hoc comparisons were conducted using the Dunn-Bonferroni method to further elucidate differences between those with differing HIFT experience and competition level. Pearson’s correlations were used to examine potential relationships between the EMI-2 variables and Competition level (none, Online, Second Round, Finals). Statistical analyses were conducted using SPPS 24.0 for Windows (SPPS, Chicago, IL), with a *p*<0.05 denoting significance (S1 Appendix).

## Results

### Survey

A total of 838 clicks on the survey link were recorded by the Bitly application. Of those, 744 individuals completed the survey, an estimated 89% response rate. However, of those who completed the survey, 22 did not provide complete data and were removed from the final analysis. A total of 722 (46.8% females) individual responses were analyzed with participants having a mean age of 32.4 ± 8.2 years (range 18–66 years; see Table 1). Although the survey was open to anyone with internet access and participating in HIFT, the majority of respondents reported living in the United States (92%).

**Table 1:** Participant characteristics.

	< 6 months (*n* = 111)	6–12 months (*n* = 142)	1–2 years (*n* = 222)	3–5 years (*n* = 209)	> 5 Years (*n* = 38)	Overall (*n* = 722)
**Age (*M±SD*)**	31.3***±***9.3	31.7***±***8.8	32.6***±***7.8	33.2***±***7.7	33.4***±±***6.2	32.4***±***8.2
**Sex (*% Female*)**	55.9	48.3	52.5	37.8	28.9	46.8
**BMI (*M±SD*)**	25.9***±***4.1	25.6***±***4.4	25.6***±***3.8	25.6***±***3.8	25.7***±***3.5	25.6***±***4.1
*Competition Level*						
***Online (n = 330*, *41% Female)***	0.9%	11.3%	51.8%	78.0%	78.9%	45.7%
***Second Round (n = 75*, *48% Female)***	0.9%	1.5%	11.5%	22.5%	26.3%	10.4%
***Finals (n = 26*, *42% Female)***	0.0%	0.0%	0.9%	8.1%	18.4%	3.6%

Note: Participants are categorized by the highest level in which they competed, as individuals who competed in the Finals also competed in the Online qualifier and Second round.

### Differences in motives between years of participation

A MANOVA revealed significant differences between length of participation and participatory motives (Wilk’s λ = 0.830, *F*(5, 729) = 1.947, *p*< 0.001, *η*^*2*^_*p*_ = 0.037). Specifically, the motivational factors of revitalization (*F*(5,722) = 2.761, *p* = 0.018, *η*^*2*^_*p*_ = 0.019), enjoyment (*F*(5,722) = 3.704, *p* = 0.003, *η*^*2*^_*p*_ = 0.025), affiliation (*F*(5,722) = 3.587, *p* = 0.003, *η*^*2*^_*p*_ = 0.024), competition (*F*(5,722) = 3.896, *p* = 0.002, *η*^*2*^_*p*_ = 0.026), and weight management (*F*(5,722) = 6.845, *p*< 0.001, *η*^*2*^_*p*_ = 0.045) all differed by length of participation. Post-hoc analysis revealed significant differences (*p*_*s*_< 0.038) between motivational factors, where individuals with a longer length of participation, especially >3 years, scored higher than those with a shorter time participating (<6 months) on enjoyment, affiliation, and competition, while those having the shortest participation length scored highest in the weight management motive (see Table 2). No other statistically significant differences were observed between motivational factors and length of participation.

**Table 2:** Participatory motives across length of participation (M ± SD, n = 722).

	Length of Participation in HIFT					
	< 6 months	6–12 months	1–3 years	3–5 years	> 5 years	*p-*value
**Intrapersonal**						
Stress Management		3.6±1.1	3.7±1.1	3.8±1.0	3.8±1.1	3.8±1.0	0.275
Revitalization	4.0±1.0	4.2±0.8	4.2±0.7	4.3±0.7*	4.2±0.8	0.027
Enjoyment	4.0±1.1	4.2±0.9	4.3±0.8*	4.4±0.7*	4.4±0.6	0.006
Challenge	4.0±1.1	3.8±1.0	3.8±1.0	3.9±0.9	3.9±0.7	0.668
**Interpersonal**						
Social Recognition	2.6±1.3	2.6±1.3	2.6±1.3	2.7±1.3	2.8±1.3	0.516
Affiliation	3.1±1.3	3.4±1.3	3.5±1.2*	3.6±1.1*	3.5±0.9	0.010
Competition	3.3±1.4^†^	3.2±1.5^†^	3.3±1.5^†^	3.7±1.2	3.7±1.4	0.003
**Health-related**						
Health Pressures	1.6±1.2	1.7±1.1	1.6±1.2	1.7±1.1	1.7±1.1	0.607
Ill-health Avoidance	3.8±1.1	4.0±1.1	3.8±1.1	3.9±1.0	3.9±1.0	0.198
Positive Health	4.5±0.7	4.6±0.6	4.6±0.6	4.6±0.6	4.6±0.6	0.147
**Body-related**						
Weight Management	3.6±1.3	3.4±1.4	3.1±1.4*	2.8±1.4*	2.6±1.1*	<0.001
Appearance	3.6±1.0	3.7±0.9	3.7±0.9	3.5±1.1	3.5±1.0	0.302
**Fitness-related**						
Strength & Endurance	4.6±0.4	4.5±0.6	4.4±0.6	4.4±0.6	4.4±0.6	0.890
Nimbleness	3.9±1.0	3.9±1.0	3.8±1.0	3.9±1.0	3.8±1.0	0.952

Note

*indicates significant difference (using Dunn-Bonferroni adjustment) from < 6 months group at *p*< 0.05.

^†^indicates significant difference from 3–5 years group at *p*< 0.05.

### Differences in motives between competition levels

#### Online competition (Level 1)

A repeated measures MANOVA revealed a significant interaction between length of participation and Online competition participation (Wilk’s λ = 0.879, *F*(5,723) = 1.331, *p* = 0.035, *η*^*2*^_*p*_ = 0.026), where significant interactions were observed for the health pressures, positive health, and nimbleness motives differed (*F*_*s*_(5,722) = 2.273–2.532, *p*_*s*_ < 0.046, *η*^*2*^_*p*_ = 0.028–0.046) between length of participation and the Online competition participation. Further, significant differences in motivational factors were observed for those who had competed in the Online competition (*n* = 330; 55%) and for those who had not (*n* = 407; 45%). Specifically, the motives revitalization (*F*(1,722) = 7.293, *p* = 0.007, *η*^*2*^_*p*_ = 0.010), enjoyment (*F*(1,722) = 16.092, *p*< 0.001, *η*^*2*^_*p*_ = 0.021), challenge (*F*(1,722) = 7.189, *p* = 0.008, *η*^*2*^_*p*_ = 0.010), social recognition (*F*(1,722) = 18.266, *p*< 0.001, *η*^*2*^_*p*_ = 0.024), affiliation (*F*(1,722) = 22.621, *p*< 0.001, *η*^*2*^_*p*_ = 0.030), competition (*F*(1,722) = 48.216, *p*< 0.001, *η*^*2*^_*p*_ = 0.061), weight management (*F*(1,722) = 28.946, *p*< 0.001, *η*^*2*^_*p*_ = 0.038) differed. Individuals who had competed in the Online competition scored higher than their non-competing counterparts in revitalization, enjoyment, challenge, social recognition, affiliation, and competition, while non-competitors scored highest in the weight management motive (see Table 3).

**Table 3:** Participatory motives across HIFT competition levels (M±SD, n = 722).

		*Online Qualifier*	*Second Round*	*Finals*
**Intrapersonal**				
Stress Management	Y	3.8±1.0	3.8±1.0	3.6±0.9
	N	3.7±1.1	3.7±1.0	3.8±1.1
Revitalization	Y	4.3±0.7*	4.4±0.7	4.2±0.7
	N	4.1±0.9	4.2±0.8	4.2±0.8
Enjoyment	Y	4.4±0.7**	4.4±0.7	4.3±0.7
	N	4.2±0.9	4.3±0.8	4.3±0.8
Challenge	Y	3.9±0.9*	4.0±1.2	3.9±0.9
	N	3.7±1.0	3.8±1.0	3.8±1.0
**Interpersonal**				
Social Recognition	Y	2.8±1.2**	3.0±1.2*	2.6±1.2
	N	2.4±1.3	2.6±1.3	2.6±1.3
Affiliation	Y	3.7±1.1**	3.7±1.2	3.6±1.2
	N	3.3±1.3	3.4±1.2	3.4±1.2
Competition	Y	3.8±1.2**	4.2±1.0**	4.2±0.9*
	N	3.1±1.5	3.3±1.4	3.4±1.4
**Health-related**				
Health Pressures	Y	1.7±1.1	1.6±1.1	1.4±1.1
	N	1.6±1.1	1.6±1.1	1.7±1.1
Ill-health Avoidance	Y	3.9±1.0	3.9±1.0	3.9±1.0
	N	3.9±1.1	3.9±1.1	3.9±1.1
Positive Health	Y	4.6±0.6	4.6±0.6	4.5±0.7
	N	4.6±0.7	4.6±0.6	4.6±0.6
**Body-related**				
Weight Management	Y	2.8±1.4**	2.2±1.4**	2.1±1.3**
	N	3.4±1.4	3.2±1.4	3.1±1.4
Appearance	Y	3.6±1.0	3.4±1.1*	3.0±0.9**
	N	3.7±1.0	3.7±1.0	3.7±1.0
**Fitness-related**				
Strength & Endurance	Y	4.4±0.6	4.3±0.7	4.2±0.7*
	N	4.4±0.6	4.4±0.6	4.4±0.6
Nimbleness	Y	3.9±1.0	3.8±1.1	3.6±1.1
	N	3.8±1.0	3.9±1.0	3.9±1.0

*Note*: Y = Competed; N = Did not compete.

*Indicates significant difference (using Dunn-Bonferroni adjustment) from non-competitors at *p*< 0.05

** *p*< 0.001.

#### Second round (Level 2)

No significant interaction was observed for any motivational variables between Second round competition and length of participation (Wilk’s λ = 0.900, *F*(5,723) = 1.082, *p* = 0.295, *η*^*2*^_*p*_ = 0.021). However, for those who had competed in the Second round (*n* = 75; 10%) and for those who had not (*n* = 662; 90%), significant differences were found in the motives social recognition (*F*(1,722) = 8.072, *p* = 0.011, *η*^*2*^_*p*_ = 0.011), competition (*F*(1,722) = 24.059, *p*< 0.001, *η*^*2*^_*p*_ = 0.032), weight management (*F*(1,722) = 18.714, *p*< 0.001, *η*^*2*^_*p*_ = 0.025), and appearance (*F*(1,722) = 5.082, *p* = 0.024, *η*^*2*^_*p*_ = 0.007). Individuals who had competed in the Second round scored higher than their non-competing counterparts in social recognition and competition, while those that did not compete in the Second round scored highest in the appearance and weight management motives (see Table 3).

#### The finals (Final level)

An interaction between those who competed in the Finals and length of participation was not observed (Wilk’s λ = 0.966, *F*(5,723) = 0.898, *p* = 0.619, *η*^*2*^_*p*_ = 0.017). For those who competed in the Finals (*n* = 25; 3%), significant differences were found in the motives competition (*F*(1,722) = 4.996, *p* = 0.026, *η*^*2*^_*p*_ = 0.007), weight management (*F*(1,722) = 14.789, *p*< 0.001, *η*^*2*^_*p*_ = 0.020), appearance (*F*(1,722) = 12.631, *p*< 0.001, *η*^*2*^_*p*_ = 0.017), and strength and endurance (*F*(1,722) = 4.484, *p* = 0.035, *η*^*2*^_*p*_ = 0.006) compared to those who had not competed in the Final round (*n* = 712; 97%). Those who had competed in the Finals scored higher than their counterparts in competition, while the others scored highest in the appearance, weight management, and strength/endurance motives (see Table 3).

### Relationship between competition level and motivation

Significant relationships were found for competition level and the interpersonal (*r* = 0.230, *p*< 0.001), intrapersonal (*r* = 0.105, *p* = 0.004), and body-related (*r* = -.190, *p*< 0.001) motivation, while no relationships were found with fitness-related (*p* = 0.725) or health-related (*p* = 0.670). When examining the individual participation motives (rather than themes), social recognition, affiliation, competition, revitalization, enjoyment, and challenge had significant positive relationships with competition level (*r*_*s*_ = 0.09–0.25, *p*_s_≤ .01) while weight management and appearance had negative relationships (*r*_*s*_ = 0.08–0.22, *p*_s_< 0.03; see Table 4). No other significant relationships were found.

**Table 4:** Relationships between motivation and competition level*.

	Pearson’s *r*	*p*-value
***Health-related***		
Health Pressures	0.01	0.783
Ill Health Avoidance	0.02	0.668
Positive Health	0.01	0.751
***Interpersonal***		
Social Recognition	0.15	< .001
Affiliation	0.15	< .001
Competition	0.25	< .001
***Body-related***		
Weight Management	-0.22	< .001
Appearance	-0.03	0.029
***Intrapersonal***		
Stress Management	0.04	0.297
Revitalization	0.10	0.010
Enjoyment	0.12	0.001
Challenge	0.09	0.012
***Fitness-related***		
Strength & Endurance	-0.04	0.270
Nimbleness	0.01	0.857

Note

* highest reported level of the competition engagement.

The order is as follows: non-competitors, Open qualifier, Second Round, the Finals.

## Discussion

The purpose of this study was to examine participation motives reported by individuals actively engaged in HIFT, but with varying lengths of experience and competition levels. Our first hypothesis, that those with greater length of participation would have stronger intrinsic motivators (e.g. revitalization, enjoyment, challenge, stress management) and stronger fitness-related motives (e.g. strength and endurance, nimbleness), was partially supported. Only enjoyment was shown to be significantly lower for those with the least participation time (i.e., <6 months), although the strength of enjoyment increased with more experience. Our second hypothesis, that those with shorter length of participation would be more extrinsically motivated, was not supported. Those with greater length of participation reported greater affiliation and competition motives compared to those with less, and there were no differences for social recognition, which was not expected. Further, and regardless of length of participation, individuals who participate in HIFT reported high levels of health-related motives (e.g. ill-health avoidance, positive health), fitness-related motives, and intrapersonal motives, which have been shown to be important factors for exercise adherence [9]. Weight-management was a stronger motive to engage in HIFT for those with the shortest length of participation (<6 months), and again strength of this motive decreased with increasing participation length. Weight management was the weakest motive in those with >5 years of participation in HIFT. Finally, our third hypothesis was supported as all interpersonal motives (i.e., social recognition, affiliation, and competition) were stronger motives for those competing in the Online competition compared to the non-competitors, while body-related motives (i.e., weight management and appearance) were less important to competitors. Thus, our findings support the notion that motivation to participate in HIFT differs between those just beginning (e.g., <1 year) and those who have adhered to this unique fitness method for greater lengths of time.

### Intrapersonal-related motives

Intrapersonal-related motives refer to intrinsic motivation, satisfying the individual’s internal needs. In this study, the intrapersonal motives of enjoyment, challenge, revitalization, and stress management were fairly strong, consistent with previous work [23], where enjoyment was a stronger motive for HIFT participants compared to other fitness groups (e.g., group exercisers, individual exercisers, personal trainers). No differences in the intrapersonal motives stress management or challenge were observed between individuals with differing length of participation. This suggests that the participants had “fairly strong” to “extreme” intrapersonal motives for engagement. Interestingly, as length of participation increased in this sample, strength of enjoyment increased as well. Those with longer HIFT participation scored significantly higher on enjoyment in comparison to those with the least HIFT experience. Nonetheless, all participants reported enjoyment being an important reason to exercise, regardless of experience. As intrapersonal motives reflect intrinsic motivation, it seems that even those with the least HIFT experience tend to be intrinsically motivated to continue engagement. This supports previous findings on the importance of enjoyment for prolonged exercise adherence [8, 9].

### Interpersonal-related motives

Interpersonal-related motives consist of affiliation, competition, and social recognition motives. These motives can be considered extrinsic because they satisfy a goal that is external to the individual. In the present sample, as length of HIFT increased, strength of affiliation and competition motives increased, while social recognition did not vary with length of training. We believe that the increases in affiliation and competition motivation with greater length of participation may be the result of individuals becoming more comfortable within the gym environment, and/or an increased ability or greater self-efficacy to perform at a competitive level [33]. Simpson and colleagues [34] discussed how individuals engaged in HIFT were encouraged and motivated by the social connection and community of their gym, as well as their ability to complete particular skills, which provided increased self-efficacy to compete against other members in the gym. Similarly, Heinrich and colleagues [33] noted the important influence of social and environmental factors for individuals engaged in HIFT on exercise adherence. Thus, those with greater length of participation have potentially developed comradery and self-efficacy and are then more motivated by affiliation and the competitive atmosphere HIFT provides. Although self-efficacy could be considered an intrinsic motivator, in this context, increased self-efficacy helps facilitate friendly competition between gym members. Using the Self-Determination Theory, those that meet the basic needs of autonomy, mastery, and relatedness are said to have reached a self-determined state, and those individuals should maintain the behavior [10]. These interpersonal motives might be important factors in an individual’s relatedness, particularly affiliation. If the goal is to increase autonomy, mastery, and relatedness for individuals engaging in different forms of exercise, it may be important to note that affiliation develops with more time of participation, as interpersonal motives increased with participation years.

### Health-related motives

As a whole, this group of HIFT participants scored high for health-related motives of positive health and ill-health avoidance, regardless of experience. Health pressure was a relatively weak motive. These findings may be due to the nature of the participants who completed the survey, who were already considered regular exercisers (i.e., at least 3 months of HIFT experience). Additionally, our sample was relatively young, with a mean age of 32.4 (± 8.2) years, which may explain why these health factors were not significant between the groups, and why the health pressure motive was not that salient for any group. Previous research on participatory motives has indicated that “health pressures” is not an essential motive for younger populations [35]. Additionally, it is well known that exercise of any kind is beneficial for physical health [36]; thus, it is not surprising that the majority of participants reported the other health-related factors as strong motivators for HIFT.

### Body-related motives

For body-related motives, those with the least amount of experience reported weight management as a strong reason to engage in HIFT, while those with the greatest experience did not rate weight management as an important motivator for exercise engagement. Additionally, appearance remained similar across all HIFT participants, which was scored as a moderately strong reason to exercise. Both body-related motives (appearance, weight management) are generally considered extrinsic motives [28], and extrinsic motives do not support autonomy [37]. Our results indicate a decrease in the importance of weight management as a motive with increasing length of HIFT participation. It can be assumed, although not known, that with greater length of exercise involvement, these individuals have satisfied weight loss goals, and thus no longer find weight management as a primary motive for HIFT adherence. However, as appearance remains moderately strong across all experience levels, further information is needed to assess whether this self-reported extrinsic motive can influence self-determination or self-confidence, and further, participation adherence among this group of individuals.

### Fitness-related motives

These HIFT participants reported fitness-related motives, particularly strength and endurance, as among the strongest reasons for their exercise participation. Our findings are in agreement with those of Fisher and colleagues [23] who also reported fitness-related motives, such as strength and endurance, as important motives among HIFT participants. Moreover, fitness-related motives have been reported among various HIFT participants as being important for exercise engagement regardless of sex [23, 24]. More recently, Feito et al. [25] demonstrated that fitness related motives differed among HIFT participants dependent upon their weekly training participation, with those training more often (>5 days/week) having significantly higher fitness-related motives compared to those engaged less often (<3 days/week). Nonetheless, less is understood about the changes in exercise motivation as it relates to beginning and continuing HIFT. Thus, our findings may be a result of the constantly varied HIFT modalities, as each daily workout focuses on one or more forms of high-intensity resistance, body weight, or aerobic training. Moreover, most HIFT facilities include a daily stretching, warm-up, skill work, and cool-down period and offer flexibility training courses [38]. This focus on multiple fitness domains may be a reciprocal motivator, both for initiation and adherence in these participants.

### Mental health implications

Recognizing an individual’s motivation to initiate and maintain a fitness program, such as HIFT, could be beneficial for understanding how to best encourage them to adopt and adhere to an exercise program that should improve both their mental and physical health. The benefits from various forms of exercise, including high-intensity training, on physical health are well understood [1]. However, less is understood about the impact of high-intensity and resistance exercise forms on mental health, specifically HIFT. Regular physical activity has been repeatedly shown to be a strong preventive tool for mental diseases and disorders [1], by helping reduce symptoms of stress, anxiety, and depression [39]. Not only have studies shown that physical activity reduces symptoms of mental health disorders, but also that regular physical activity and exercise help prevent the development of some mental health diseases and disorders [2]. Gerber and colleagues [40] reported reductions in stress, depressive symptoms, subjective pain, and improvements in sleep after completing vigorous intensity exercise. However, research examining such mental health outcomes from acute bouts and interventions of vigorous/high-intensity exercise are limited. As HIFT continues to grow in popularity, studies should be carried out to better understand the impact this training modality may have on mental health outcomes.

### Limitations and future research

Although we believe our findings are novel and contribute to the literature for this topic, our study was not completed without limitations. First, our sample size (*N* = 722) is a small number of current HIFT participants [14], with the majority (92%) residing in the United States. Second, due to the inclusion criteria for the study, we did not collect data on individuals who just began this training modality. Thus, we potentially missed motivational information for individuals just adopting this new behavior, as a drop-out rate of approximately 50 percent occurs in the first 6 months [41]. Additionally, because our online survey was sent through social media and emailed to HIFT facilities, we had a convenience sample of current participants, which may have biased our results.

This study has provided a first look at participation motives of individuals actively participating in HIFT with varying length of participation and competition levels. Future studies should examine behavioral regulation (i.e., intrinsic, extrinsic, and amotivation) and barriers associated with HIFT in individuals actively engaged in, as well as those who have started but then discontinued their participation in HIFT. Such research would provide a better understanding of the different motives associated with HIFT participation and those who initiated HIFT but then chose to engage in other exercise training modalities, or none at all. Differentiating between these groups could provide a better understanding with respect to designing individualized exercise programs to promote greater participation and improved adherence, regardless of the exercise regimen. Additionally, further studies should examine what personality traits and mental health factors are associated with individuals who engage in this popular exercise form. Nevertheless, our results provide a first step in supporting the role of HIFT as a viable exercise methodology that promotes behavior change through the interaction between the individual (cognitions and behavior) and the external environment (social) by engaging in an exercise program that satisfies an individuals’ perception to change their own behavior.

## Conclusion

As past studies have provided evidence of the range of benefits in exercise engagement, these present findings provide novel information regarding changes in exercise motivation across length of participation for those engaging in HIFT. These findings suggest that even though intrinsic motives (e.g., enjoyment, revitalization, challenge) are a strong motivator for those just beginning HIFT, this trending exercise form promotes an increase in those motives with greater length of participation. Additionally, interpersonal motives (representing relatedness) also increase with participation time. As intrinsic and relatedness variables have been shown to promote exercise adherence, these findings support HIFT as an exercise form that promotes exercise behavior, prompting mental and physical health benefits.

## Supporting information

S1 Appendix Data table. (XLSX)
